# Cytokines and Chemokines as Regulators of Skeletal Muscle Inflammation: Presenting the Case of Duchenne Muscular Dystrophy

**DOI:** 10.1155/2013/540370

**Published:** 2013-11-05

**Authors:** Boel De Paepe, Jan L. De Bleecker

**Affiliations:** Laboratory for Myopathology, Department of Neurology and Neuromuscular Reference Center, Ghent University Hospital, De Pintelaan 185, 9000 Ghent, Belgium

## Abstract

Duchenne muscular dystrophy is a severe inherited muscle disease that affects 1 in 3500 boys worldwide. Infiltration of skeletal muscle by inflammatory cells is an important facet of disease pathophysiology and is strongly associated with disease severity in the individual patient. In the chronic inflammation that characterizes Duchenne muscle, cytokines and chemokines are considered essential activators and recruiters of inflammatory cells. In addition, they provide potential beneficiary effects on muscle fiber damage control and tissue regeneration. In this review, current knowledge of cytokine and chemokine expression in Duchenne muscular dystrophy and its relevant animal disease models is listed, and implications for future therapeutic avenues are discussed.

## 1. Introduction

Duchenne muscular dystrophy (DMD) is an X-linked muscle disease, with a prevalence of 1 in 3500 boys worldwide. Patients develop progressive weakness of skeletal and respiratory muscles and dilated cardiomyopathy. Clinical onset is usually between 2 and 5 years of age. Most patients loose independent ambulation in their teens, after which scoliosis develops. Death usually occurs before forty years of age and is most often the result of respiratory or cardiac failure. The biochemical cause of DMD is a severe deficiency of dystrophin, an essential component of the sarcolemmal dystrophin-associated glycoprotein complex. When complex assembly is disturbed, the linkage between the muscle cell's cytoskeleton and the extracellular matrix is compromised, leading to sarcolemmal instability and increased vulnerability to mechanical stress [1]. Fibers undergo necrosis by excessive Ca^2+^ influx [2] and are progressively replaced by connective and adipose tissue. 

The immune system plays a pivotal role in the pathogenesis of DMD. Contraction of dystrophin deficient myofibers produces severe damage and generates cycles of muscle fiber necrosis and regeneration. Necrotizing myofibers are attacked by macrophages; inflammatory cells are present throughout the endomysial, perimysial, and perivascular areas. Macrophages are the most abundant immune cells observed in DMD muscle and both proinflammatory M1 phenotype macrophages and regeneration-focussed M2 phenotype macrophages are present. Within the inflammatory areas, few T cells, B cells, and dendritic cells are also present. Infiltrating T cells are predominantly CD4+, and smaller numbers of CD8+ T cells can be found [3]. The T cell receptor repertoire of CD4+ and CD8+ T cells does not display dominant V*α* or V*β* rearrangements, which points toward a nonspecific cell recruitment to sites of muscle fiber destruction [4]. In addition to their involvement in muscle damage, T cells also play an important role in the fibrotic processes present in dystrophic muscle. T cell deficiency significantly reduces collagen matrix accumulation in the murine disease model [5]. The underlying mechanisms are complex and rely on the interplay of immune cells and cytokines [6]. 

The build up of the inflammatory response is complexly regulated through interactions between adhesion molecules, receptors, and soluble factors, recruiting immune cells from the blood stream to the muscle tissue [7]. 

## 2. Animal Models of DMD

In the last decade, improved genetic testing has made diagnostic muscle biopsies redundant in most cases, which means that nowadays DMD muscle samples only rarely become available for pathological research. It is therefore even more imperative to investigate animal models to gain insight into human disease. This is a feasible approach, as the dystrophin-associated protein complex is evolutionary ancient and highly conserved among species. By far the most studied model for DMD is the murine mdx model. Mdx mice have a premature stop codon in the dystrophin gene, which leads to the loss of functional protein. One should however remain cautious when extrapolating data obtained in the mdx model to human disease. The clinical phenotype of mdx mice is less severe and follows a different time course than human disease. Also, of importance in the context of this review, there are notable differences in the cytokine system of mouse compared to man. Dystrophin-deficient dogs seem to more closely mimic human disease, for example, the severe myopathy in golden retriever muscular dystrophy (GRMD) [8]. Dystrophin-deficient hypertrophic feline muscular dystrophy (HFMD) is characterized by early disease onset and continuous muscle fiber regeneration in the absence of significant inflammatory infiltration or proliferation of connective or adipose tissue. Some HFMD-affected cats develop cardiomyopathy [9]. Recently, zebrafish with mutations in the sapje locus containing the dystrophin gene have become available. Zebrafish embryos represent a convenient model to study disease [10] and are extremely suited to first-line drug screening [11]. It is to be expected that studies in DMD disease models, addressing the underlying disease mechanisms as well as therapeutic efficiencies, will continue to proliferate in the near future.

## 3. Cytokines

Initially, no distinct pattern of cytokine expression could be shown for DMD [12], but since then several inflammatory factors have been reported to preferentially associate with the disease [13].

### 3.1. TNF Family of Cytokines

**Figure 1:**
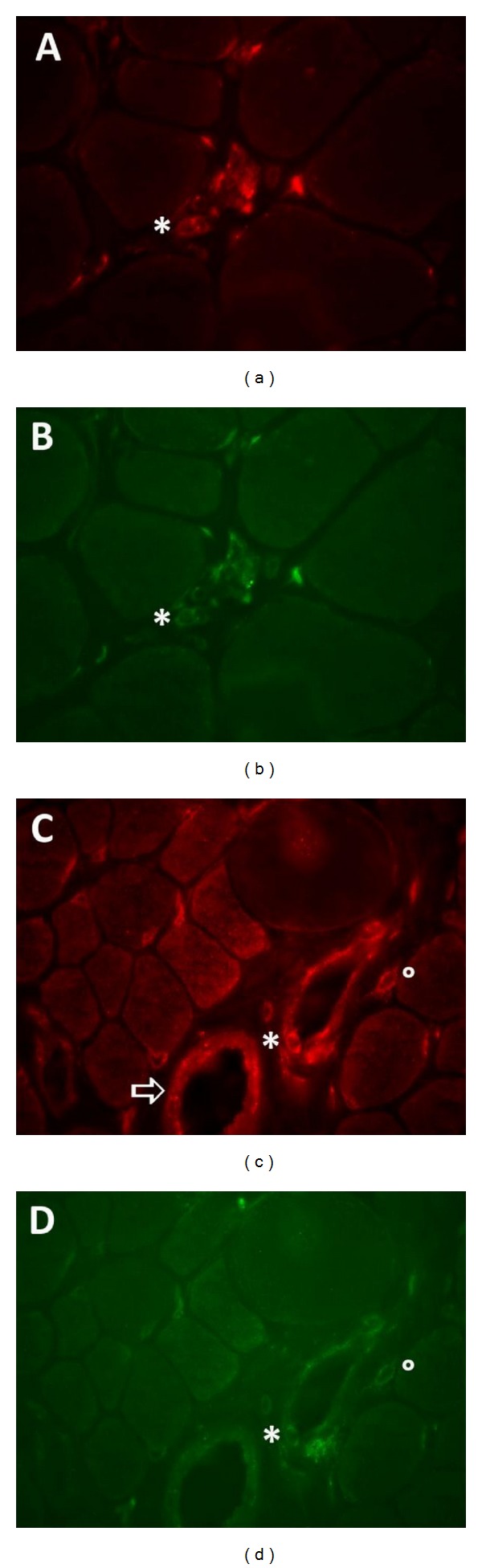
Immunofluorescent detection of TNF-*α* and IFN-*γ* in Duchenne muscular dystrophy. (a)-(b) Muscle biopsy taken from an 8-year-old patient with Duchenne muscular dystrophy caused by duplication of dystrophin exon 2, resulting in severe muscle damage, few groups of revertant fibers, and strong utrophin staining. TNF-*α* (red in (a)) is detected in a small cluster of inflammatory cells and colocalizes with CD3 (green in (b)). The asterisk is an indicative that helps to identify an individual TNF-*α*+ T cell. (c)-(d) Muscle biopsy taken from a 2-year-old patient with Duchenne muscular dystrophy caused by c.5299-5302dupATTT in dystrophin exon 37. Myopathological evaluation of the biopsy described definite muscle damage, few groups of revertant fibers, and strong utrophin staining. IFN-*γ* (red in (c)) is strongly expressed on the blood vessel endothelium (arrow) and on perivascular CD3+ T cells (green in (d)). Highlighted are an IFN-*γ*+ CD3+ T cell attached to the luminal side of the blood vessel (asterisk) and an interstitial IFN-*γ*+ CD3+ T cell (circle).

The proinflammatory members of the tumor necrosis factor (TNF) family are important regulators of chronic inflammation. TNF-*α* (TNFSF2), the prototypic catabolic cytokine and most studied member of the TNF-family, is associated with helper T cell type-1-(Th1-) mediated cellular immunity. TNF-*α* is upregulated in DMD sera [14] with levels increased 1000 times in comparison to levels in healthy subjects [15]. TNF-*α* mRNA expression is significantly higher in circulating lymphocytes of DMD patients compared to controls [16]. In DMD skeletal muscle tissues, a proportion of muscle fibers are TNF-*α* immunoreactive [17] most of which are regenerating muscle fibers [18]. However, the primary source of TNF-*α* in DMD muscle is the inflammatory cells (Figure 1(a)) that, by doing so, further perpetuate the inflammatory response. Diaphragm of mdx mice contains significantly higher TNF-*α* mRNA levels than controls [19], and TNF-*α* protein strongly colocalizes with tissue infiltrating macrophages [20]. In contrast to what was expected, TNF-*α* knockout mdx mice do not exhibit an amelioration of muscle pathology [21], adding nuance to the considered destructive role of TNF-*α* in muscle dystrophy. Lymphotoxin-*β* (LT-*β*; TNFSF3) is a key factor in lymphoneogenesis and, through the expression of adhesion molecules, cytokines and chemokines, it regulates innate and adaptive immune responses. LT-*β* protein levels are significantly upregulated in muscular dystrophies, compared to normal skeletal muscle. Blood vessels and the sarcolemma of DMD fibers are LT-*β* positive, and staining is further enhanced in the regenerating fibers, sometimes accompanied with sarcoplasmic staining [22]. LT-*β* expressed by muscle fibers could serve as an anchor point to attract inflammatory cells to the tissue sites.

 Muscle fiber necrosis, an accidental form of cell death triggered by physical tissue damage, is an abundant phenomenon in DMD. However, regulated forms of cell death could alternatively be involved in muscle damage development. Recently, a regulated form of necrosis, which can be initiated by TNF-*α*-induced receptor-interacting protein kinase activity, has been recognized [23]. In addition, the well-characterized process of apoptosis follows a series of programmed events, relying upon regulated expression of specific proteins that signal cells to their death. DNA fragmentation and changes in cell structure characteristic to apoptotic processes can be observed in soleus muscle from mdx mice [24]. Also, the percentage of apoptotic nuclei is higher in DMD muscle than in controls [25]. Several TNF cytokine family members are actively involved in apoptosis. FasL (TNFSF6) has been shown to induce muscle cell apoptosis *in vitro* [26]. FasL mRNA expression is significantly higher in peripheral blood lymphocytes of DMD patients compared to controls [16]. A small proportion of DMD muscle fibers express the corresponding receptor Fas [27]. Induction of both ligand and receptor could, unlike in idiopathic inflammatory myopathies [28], indicate an involvement of Fas/FasL—mediated apoptosis in DMD muscle atrophy and degeneration. TNF-like weak inducer of apoptosis (TWEAK; TNFSF12) is a major inducer of muscle wasting [29] and preventer of muscle regeneration [30]. To our knowledge, no data is available at this moment regarding TWEAK expression in DMD.

### 3.2. Interleukins

Interleukins (IL), of which 36 different forms have been identified so far, play a major role in the immune system. Most important data available at this moment will be discussed under this heading, except for IL-8 which will be discussed in the chemokine section. 

 The involvement of proinflammatory IL-1 in muscular dystrophy remains a topic of debate. Neither IL-1*α* nor IL-1*β* immunoreactivity could be shown in a study investigating 8 DMD muscle samples [17], and the IL-1 family has been reported downregulated in DMD serum [31]. However, another study describes IL-1*β* as being increased in DMD muscle [32]. Also, diaphragm of mdx mice contains significantly higher IL-1*β* mRNA levels than control mice [19], and IL-1*β* protein colocalizes with the infiltrating macrophages [20].

 IL-6 is a cytokine with both proinflammatory and antiinflammatory properties. It is a helper T cell type 2 (Th2) cytokine, meaning that it promotes IgE and eosinophilic responses in atropy and counteracts Th1-driven proinflammatory responses. On the other hand, IL-6 exhibits proinflammatory activity through activation of the transcription factor nuclear factor *κ*B. IL-6 concentrations are significantly higher in serum of DMD patients (3.77 ± 2.71 pg/mL) compared to healthy age-matched controls (1.93 ± 1.38 pg/mL) [31] and follow the disease time-course [32]. In DMD muscle, IL-6 mRNA levels display a significant increase compared to controls. The level increases with age: from 26-fold at age 4 years to 148-fold between 5 and 9 years [33]. Blocking IL-6 through injection with a monoclonal antibody causes an increase of muscle inflammation in the mdx mouse model, further suggesting an anti-inflammatory effect, possibly by mediating muscle repair [34]. 

 IL-10 functions as a suppressor of inflammation through its differential effect on the different macrophage subtypes: deactivating M1 macrophages and activating the M2 phenotype. M1 macrophages function within the Th1 response and produce copious amounts of proinflammatory cytokines, while M2 macrophages promote angiogenesis and tissue repair and remodeling [35], a phenomenon also present in muscle [36]. IL-10 prevents the production of Th1-associated cytokines such as TNF-*α* and IFN-*γ* [37]. Its expression is 8 to 15-fold increased in mdx quadriceps compared with wild type muscle, possibly as a protective reflex of the tissue. IL-10 null mutation causes severe reduction of muscle strength due to an imbalance between M1 and M2 macrophages [38].

 IL-15 has proinflammatory characteristics as a stimulator of T cell proliferation and NK-activity but can also be of benefit to tissue recovery by increasing myogenic differentiation [39]. Mdx diaphragm contains some IL-15 reactivity especially in proximity of inflammatory cells. Treatment with recombinant IL-15 has a mild anabolic effect on diaphragm function [40]. 

 IL-17 is a potent amplifier of ongoing inflammation and plays an important role in the progression of chronic inflammation and autoimmunity. IL-17 induces TNF-*α*, IL-1*β*, and IL-6 expression and stimulates the production of chemokines such as CXCL1, CXCL5, IL-8, CCL2, and CCL7 [41]. In DMD quadriceps muscle IL-17 mRNA is induced while being undetectable in control muscle, and expression is associated with functional outcome at 6 years of age [42].

### 3.3. Interferons

The interferon (IFN) family members are divided among three classes. Type I IFN (in humans IFN-*α*, IFN-*β*, and IFN-*ω*) are associated with innate immunity. The sole human IFN type II is the proinflammatory Th1 cytokine IFN-*γ*. IFN-*γ* expression is elevated in mdx muscle in the early disease stage when many M1 macrophages are present [43]. In the regenerating stage of disease, IFN-*γ* ablation causes a significant reduction in muscle fiber injury, an effect probably mediated through the observed shift in favor of M2 phenotype macrophages [44]. In DMD muscle, the strongest IFN-*γ* expression is observed in the blood vessel endothelial cells and in interstitial T cells (Figure 1(c)).

### 3.4. Transforming Growth Factors

Transforming growth factors (TGF) are a large group of cytokines that include TGF-*β* 1 to 3 and myostatin. Several members of the TGF family play important roles as regulators of skeletal muscle homeostasis and have been implicated in inherited and acquired muscle disorders. 

 TGF-*β* is a pleiotropic cytokine with important roles in inflammation, cell growth, and tissue repair [45]. TGF-*β* is a fibrogenic cytokine that induces synthesis and accumulation of extracellular matrix components. In adult muscle, TGF-*β* negatively affects skeletal muscle regeneration by inhibiting satellite cell proliferation and myofiber fusion. TGF-*β*1 mRNA levels are significantly higher in 30-day-old GRMD than in healthy dogs. TGF-*β* immunoreactivity is mostly confined to the connective tissue and varies between individual animals. Interestingly, in adult GRMD dogs TGF-*β* mRNA levels decrease to levels lower than those in normal dogs [46]. TGF-*β*1 activation appears to be associated with muscle wasting in DMD. TGF-*β*1 expression, mostly originating from muscle resident fibroblasts, is most pronounced in the early stages of muscle fibrosis and peaks between 2 and 6 years of age [47]. Fibroblasts from DMD muscle biopsies differ from control fibroblasts, displaying higher rates of proliferation. In addition, DMD muscle-derived fibroblasts contain significantly higher TGF-*β* protein levels, though similar levels of TGF-*β*1 mRNA are present as in controls [48]. Of special interest to determine patient prognosis is that a haplotype of the latent TGF-*β* binding protein 4 gene has been shown to correlate with prolonged ambulation of DMD patients [49]. 

 Skeletal muscle specific myostatin (TGF-*β*8) is an inhibitor of muscle growth. Mdx mice lacking myostatin display less fibrosis in the diaphragm and are stronger and more muscular than their normal mdx counterparts [50]. Another study found that myostatin is downregulated in muscle from DMD infants as well as symptomatic patients [51] indicating that this pathway may contribute less to muscle wasting in human disease. Myofibroblasts prepared from DMD biopsies, however, have been shown to express significantly higher myostatin mRNA levels than controls [48]. 

## 4. Chemokines

**Figure 2:**
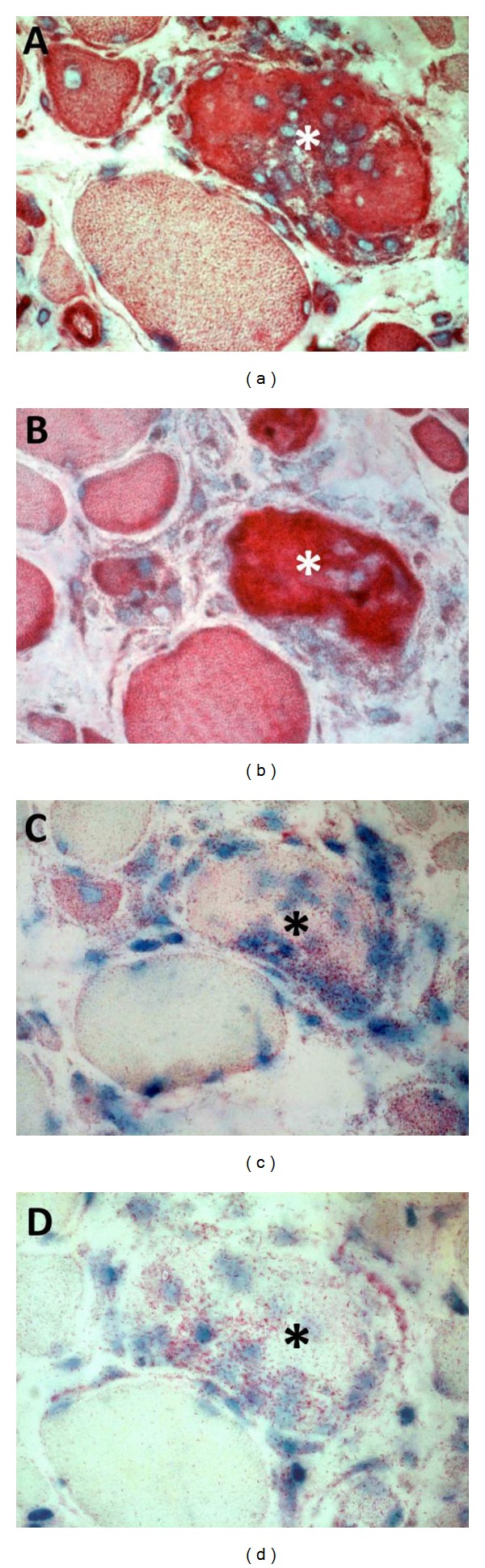
Chemokine staining in Duchenne muscular dystrophy. Nonconsecutive sections showing the same microscopic field containing a necrotic muscle fiber invaded by macrophages (asterisk). The muscle biopsy was taken from an 8-year-old patient with Duchenne muscular dystrophy caused by duplication of dystrophin exon 2. Upon diagnostic myopathological evaluation, the biopsy displayed severe muscle damage, few groups of revertant fibers, and strong utrophin staining. Chemokines were immunostained and visualized with a secondary antibody using the streptavidin-biotin labeling system and 3-amino-9-ethylcarbazole chromogen (Dako, Glostrup, Denmark). Cell nuclei were counterstained with hematoxylin (blue). The sarcoplasm of a necrotic fiber is strongly positive for CXCL8 (red in (a)) and CXCL11 (red in (b)). The cytoplasm of the necrotic fiber and its invading inflammatory cells are moderately positive for CCL5 (red in (c)) and faintly positive for CCL17 (red in (d)). Small regenerating fibers stain for all four chemokines with varying intensities.

Chemotactic cytokines or chemokines are subdivided into families according to their primary structure (most belong to the alpha (CXCL) or beta (CCL) families) and exert their biological functions by binding to G protein-coupled receptors [52]. Chemokines interact with other cytokines and adhesion molecules and their activities go way further than the attraction of leukocytes to inflammatory sites. CCL17, for instance, has been shown to enhance tissue fibrosis [53]. While the chemokine expression profile in healthy skeletal muscle is fairly limited, many chemokines are induced or upregulated in dystrophic muscle (Tables 1 and 2) [54–58]. The individual muscle tissue distribution of some chemokines has been determined and shows that they can differentially be allocated to inflammatory cells, blood vessel endothelium, and/or the muscle fibers themselves (Figure 2). Three chemokines, being CXCL8 (IL-8), CCL2, and CCL5, come forward as possible effectors of the cytotoxic activities of M1 macrophages in DMD. CCL2 upregulation in particular seems an early event in muscle dystrophy, present in DMD before the age of 2 years [55] and detectable in 14-day old mdx mice [57].

## 5. Comparison with Myositis of Other Origins

Muscular dystrophies are a clinically, biochemically, and genetically heterogeneous group of disorders [59]. Dystrophin mutations are not just responsible for DMD but also cause a spectrum of other X-linked conditions, such as the milder Becker muscular dystrophy (BMD), cardiomyopathies, and mental retardation. Also, defects in other dystrophin-associated proteins cause disease, including autosomal recessive inherited limb-girdle muscular dystrophies. In many subtypes, muscle inflammation and muscle wasting contribute to disease progression, potentially implicating cytokines and chemokines in their pathogenesis. For instance, the presence of endomysial and perivascular inflammation is an established hallmark of dysferlinopathy [60]. 

 In contrast to the relatively limited amount of published DMD data, a multitude of reports is available on the expression of cytokines in the different idiopathic inflammatory myopathies (IIM), which include dermatomyositis (DM), polymyositis (PM) and sporadic inclusion body myositis (IBM) [61]. This allows for some comparison between primary muscle inflammation in IIM and dystrophy-associated muscle inflammation. In a single BMD patient included in a multiplex cytokine immunoassay study, CCL2 levels are 11 pg/mg muscle protein, while those in 6 patients per IIM group were 45 ± 51 (DM), 15 ± 9 (PM) and 13 ± 9 (IBM), respectively [62]. In DMD quadriceps muscle, TNF-*α*, IL-6, and CCL2 mRNA levels are lower than in juvenile DM [42]. The observed more moderate expression levels could be indicative to the secondary nature of muscle inflammation as opposed to the primary inflammatory origin of the IIM. Although there unmistakably are universal inflammatory processes at hand, data also point to specific roles for cytokines and chemokines in DMD. The expression profiles of M1 macrophages are peculiar when DMD and IIM are compared. Also, in IIM strong expression of CXCR3 is observed on the muscle infiltrating T cells, indicating their involvement in Th1 immune responses. Such polarization of T cells is less obvious in DMD muscle. In fact, the muscle infiltrating T cells in DMD express a strikingly limited repertoire of chemokines in comparison to their IIM counterparts [54]. 

## 6. Relevance to DMD Disease Management

The medical community still awaits the coming of age of molecular dystrophin salvaging therapies [63]. In this respect, exon skipping [64] and suppression of stop codons [65] are considered strategies of increasing functional dystrophin expression. However, surfacing results of clinical trials, more particular those using AAV-mediated delivery of mini-dystrophin, are suggestive of important acquisition of T cell immunity targeting the dystrophin protein [66]. Earlier, it had been postulated that such priming was unlikely, due to the presence of revertant fibers in many patients which would theoretically safeguard dystrophin replacement from the immune system. Nonetheless, it is becoming more and more obvious that monitoring of cellular immune responses will be a priority in all ongoing and future experimental therapies aimed at increasing the number of dystrophin positive muscle fibers. A recent study demonstrated that circulating dystrophin primed T cells are frequently encountered in DMD, increased with age, and reduced by glucocorticoid therapy [67]. 

 Immunosuppression, administering glucocorticoids in particular, remains standard treatment for DMD today. Although anti-inflammatory therapy may add years to DMD patient ambulation, steroids are associated with important adverse effects [68]. The characterization of the factors that drive inflammation and guide specific subsets of leukocytes to the tissues raises hopes of attempting more selective immunomodulatory intervention. Strategies aimed at neutralizing individual cytokines or chemokines could be an amenable approach to reduce side effects. 

### 6.1. Targeting the Culprits While Sparing the Protectors

Specifically targeting cytokines and chemokines with predominant proinflammatory activities, such as TNF-*α*, is under exploration. The TNF-*α* neutralizing antibody infliximab delays and reduces muscle damage in mdx mice [69]. Soluble TNF-receptor etanercept, a dimeric fusion protein composed of an extracellular ligand-binding portion of the human p75 TNF-receptor linked to the Fc portion of human IgG, reduces muscle fibrosis [70] and necrosis [71]. The disruption of chemokine-mediated signaling also seems, at first glance, an attractive therapeutic possibility. An approach could be to selectively block a chemokine receptor with a key catabolic role by either a small-molecule antagonist, antibody, binding protein, or protein agonist [72]. Several chemokine-receptor antibodies are entering the clinic, including an anti-CCR2 monoclonal antibody named MLN1202 (Millenium Pharmaceuticals, Cambridge, MA, USA) currently being tried for various inflammatory diseases. However, strategies targeting the chemokine system present with certain inherent difficulties. Firstly, several chemokines are up-regulated in DMD. The redundancy of function of part of them makes it difficult to design effective therapeutic interventions. Secondly, there could be considerable inter-patient variability, as well as differences between the stages of the disease. More research is necessary to address these issues. Thirdly, chemokines can have benefits for tissue recovery, by activating muscle fiber regeneration and recruiting non-cytotoxic macrophage subpopulations that stimulate muscle tissue rebuilding [73]. For instance, when considering the anti-CCR2 avenue, its ligand CCL2 has the potential to drive forward chronic inflammation, but the importance of CCL2 in muscle regeneration has also been recognized [74, 75]. 

 In addition, strategies aimed at neutralizing fibrogenic cytokines or cytokines associated with muscle wasting are under exploration for treating DMD. For instance, the TGF-*β*1 antagonist pirfenidone improves cardiac function in mdx mice [76]. The TGF-*β* blocker suramin decreases fibrosis and offers benefit in grip strength in mdx mice [77]. A TGF-*β* neutralizing antibody decreases fibrosis and improves regeneration in mdx mice [78]. Inhibition of myostatin with a neutralizing antibody [79], soluble decoy receptor [80], or myostatin binding propeptide [81] has also been put forward. An *in vitro* model, using nodules of DMD muscle-derived fibroblasts grown onto a solid substrate, has been developed which allows convenient screening of potential antifibrotic agents [82]. 

### 6.2. Reprogramming the Immune Response

While M1 macrophages have a destructive cytokine repertoire, the M2 phenotype promotes angiogenesis, tissue repair, and remodeling. In mdx muscle, M1 macrophages predominate during the early, acute stage. The balance tips over to the M2 phenotype in the regenerative and progressive phase of the disease. In other words, the M1/M2 balance evolves beneficially with M1 macrophages undergoing deactivation as the disease progresses from the acute necrotic to the regenerative phase. M1 density significantly reduces with age in mdx soleus (4 versus 12 weeks) [43]. This could account for the milder disease phenotype of mdx mice compared to human disease, as in contrast percentages of M1 and M2 phenotype macrophages seem strikingly constant in DMD muscle taken at different disease stages [54]. Therapeutic agents regulating the M1/M2 balance in favor of the M2 phenotype, such as cannabinoid CB2 receptor agonists, could be of benefit to patients [83]. Interestingly, glucocorticoids as such have also been shown to favor a shift of macrophage phenotype, reducing the numbers of M1 macrophages by half in patients treated with prednisone (0.75 mg/kg/day) during 6 months [84].

## 7. Conclusions

In dystrophic skeletal muscle, part of the accumulating muscle damage is caused by ongoing activation of inflammatory cells rather than by direct mechanical damage. Current knowledge, of which a large part is summarized in this review, supports an important and diversified role for cytokines and chemokines in the DMD-associated muscle inflammation. The fact that a number of chemokines are expressed directly by the muscle fibers suggests that the tissue itself contributes to the chemotaxic process, actively perpetuating the chronic inflammation.

## Figures and Tables

**Table 1:** Alpha-chemokine expression in Duchenne muscular dystrophy and its mouse model.

Systematic name	Common name	Tissue	mRNA quantity	Protein quantity	Protein localization	Reference
CXCL1	GRO-alpha	DMD quadriceps muscles			BV, MF, M*φ*, T, DC	[54]
CXCL2	GRO-beta	DMD quadriceps muscles			BV, MF, M*φ*	[54]
CXCL3	GRO-gamma	DMD quadriceps muscles			BV, MF, M*φ*, DC	[54]
CXCL8	IL-8	DMD quadriceps muscles			BV, MF, M*φ*	[54]
CXCL10	IP-10	DMD quadriceps muscles			BV, (MF), M*φ*, T	[54]
CXCL11	ITAC	DMD quadriceps muscles			BV, (MF), M*φ*	[54]
CXCL12	SDF-1	DMD quadriceps muscles	Increased 2.3x			[55]
		DMD quadriceps muscles			BV, MF	[54]
		DMD serum		Increased 1.2x		[56]
CXCL14	BRAK	mdx hindlimb muscles	Increased 1.7x			[57]

Breast and kidney derived (BRAK); blood vessel (BV), alpha-chemokine (CXCL), dendritic cell (DC), Duchenne mouse model (mdx), growth related oncogene (GRO), interleukin 8 (IL-8), interferon-inducible protein of 10 kd (IP-10), interferon-inducible T cell alpha chemo-attractant (ITAC), muscle fiber (MF), macrophage (M*φ*), stromal cell-derived factor (SDF), T cell (T). Rare observations are indicated between brackets.

**Table 2:** Beta-chemokine expression in Duchenne muscular dystrophy and its mouse model.

Systematic name	Common name	Tissue	mRNA quantity	Protein quantity	Protein localization	Reference
CCL2	MCP-1	mdx hindlimb muscles	Increased 62.7x	Increased 4.1x	MF, M*φ*	[57]
		DMD quadriceps muscles	Increased 1.4x			[55]
					BV, M*φ*	[54]
CCL3	MIP-1 alpha	mdx diaphragm	Increased			[58]
CCL5	RANTES	mdx hindlimb muscles		Increased 2.3x		[57]
		mdx diaphragm	Increased	Increased		[58]
		DMD quadriceps muscles			M*φ*	[54]
CCL7	MCP-3	mdx hindlimb muscles	Increased 14.7x			[57]
		DMD quadriceps muscles			M*φ*	[54]
CCL8	MCP-2	mdx hindlimb muscles	Increased 28.9x			[57]
CCL9	MIP-1 gamma	mdx hindlimb muscles	Increased 7.9x	Increased 2.4x		[57]
CCL11	eotaxin	mdx hindlimb muscles		Increased 2.0x		[57]
CCL17	TARC	DMD quadriceps muscles			(M*φ*)	[54]

Blood vessel (BV), beta-chemokine (CCL), Duchenne muscular dystrophy (DMD), monocyte chemoattractant protein (MCP), Duchenne mouse model (mdx), muscle fiber (MF), macrophage (M*φ*), macrophage inflammatory protein (MIP), regulated upon activation, normal T cell expressed and secreted (RANTES), thymus and activation-regulated chemokine (TARC). Rare observations are indicated between brackets.

## Abbreviations

BMD: Becker muscular dystrophy
CCL: Beta-chemokine
CXCL: Alpha-chemokine
DMD: Duchenne muscular dystrophy
GRMD: Golden retriever muscular dystrophy
HFMD: Hypertrophic feline muscular dystrophy
IIM: Idiopathic inflammatory myopathies
IFN: Interferon
IL: Interleukin
TGF: Transforming growth factor
TNF: Tumor necrosis factor.
